# Combination of whole genome sequencing and supervised machine learning provides unambiguous identification of *eae*-positive Shiga toxin-producing *Escherichia coli*

**DOI:** 10.3389/fmicb.2023.1118158

**Published:** 2023-05-12

**Authors:** Fabien Vorimore, Sandra Jaudou, Mai-Lan Tran, Hugues Richard, Patrick Fach, Sabine Delannoy

**Affiliations:** ^1^ANSES, Laboratory for Food Safety, Genomics Platform IdentyPath, Maisons-Alfort, France; ^2^ANSES, Laboratory for Food Safety, COLiPATH Unit, Maisons-Alfort, France; ^3^Bioinformatics Unit, Genome Competence Center (MF1), Robert Koch Institute, Berlin, Germany

**Keywords:** machine learning, Shiga toxin-producing *Escherichia coli*, food safety, metagenomics, raw milk

## Abstract

**Introduction:**

The objective of this study was to develop, using a genome wide machine learning approach, an unambiguous model to predict the presence of highly pathogenic STEC in *E. coli* reads assemblies derived from complex samples containing potentially multiple *E. coli* strains. Our approach has taken into account the high genomic plasticity of *E. coli* and utilized the stratification of STEC and *E. coli* pathogroups classification based on the serotype and virulence factors to identify specific combinations of biomarkers for improved characterization of *eae*-positive STEC (also named EHEC for enterohemorrhagic *E.coli*) which are associated with bloody diarrhea and hemolytic uremic syndrome (HUS) in human.

**Methods:**

The Machine Learning (ML) approach was used in this study on a large curated dataset composed of 1,493 *E. coli* genome sequences and 1,178 Coding Sequences (CDS). Feature selection has been performed using eight classification algorithms, resulting in a reduction of the number of CDS to six. From this reduced dataset, the eight ML models were trained with hyper-parameter tuning and cross-validation steps.

**Results and discussion:**

It is remarkable that only using these six genes, EHEC can be clearly identified from *E. coli* read assemblies obtained from in silico mixtures and complex samples such as milk metagenomes. These various combinations of discriminative biomarkers can be implemented as novel marker genes for the unambiguous EHEC characterization from different *E. coli* strains mixtures as well as from raw milk metagenomes.

## 1. Introduction

Shiga toxin-producing *Escherichia coli* (STEC) are important zoonotic pathogens comprising more than 400 serotypes (Beutin and Fach, 2015). Pathogenic STEC strains such as enterohemorrhagic *E. coli* (EHEC) may cause hemorrhagic colitis (HC) and hemolytic-uremic syndrome (HUS) in humans. However, it remains difficult to fully define human pathogenic STEC or identify virulence factors for STEC that clearly foresee their capacity to cause human disease (European Food Safety Authority and European Centre for Disease Prevention and Control, 2021). The production of Shiga toxin (*stx* genes) by highly pathogenic STEC (i.e., EHEC) is the major virulence factor responsible for HUS, but many *E. coli* strains that produce Shiga toxin do not cause HUS. Therefore, the identification of virulent STEC strains based solely on the presence of *stx* genes may be misleading. Shiga toxins comprise a growing family of genes with a vast type diversity (Scheutz et al., 2012). The Stx family splits into two major branches, Stx1 and Stx2, which are immunologically not cross-reactive and show about 55% difference in their amino acid sequences (Müthing et al., 2009). In addition to producing one or both types of Shiga toxin, typical EHEC strains harbor a genomic pathogenicity, called the “locus of enterocyte effacement” (LEE). This locus was first identified in enteropathogenic *E. coli* (EPEC), a leading cause of infant diarrhea in developing countries. The LEE carries genes encoding proteins involved in the pathogenicity of *E. coli* strains, as they participate in bacterial colonization of the gut and destruction of the intestinal mucosa (Nataro and Kaper, 1998). For example, the intimin-encoding gene (*eae*) is directly involved in the attaching and effacing (A/E) process and serves as an indicator for the A/E function in the bacteria (Zhang et al., 2002). As mentioned above, prediction of STEC pathogenicity using available markers is challenging, but strains positive for Shiga toxin (in particular the *stx2* genes) and *eae* (intimin production) genes have been shown to be associated with a higher risk of causing more severe illness than other virulence factor combinations (European Food Safety Authority, 2007, 2013). STEC are traditionally considered to be zoonotic pathogens that are primarily food- and water-borne, with the main reservoir being the digestive tract of mammals, particularly ruminants (Gill et al., 2022). Consumption of contaminated food, such as undercooked ground meat and unpasteurized dairy products, is the principal source of infection. Current methods for EHEC identification in feed and food samples rely on the molecular detection of *stx, eae*, and the top five or top seven EHEC serogroups, followed by strain isolation, as described in the ISO/TS 13136:2012 (EU) and MLG5C.02 (US) reference methods (International Organization for Standardization, 2012; European Food Safety Authority and European Centre for Disease Prevention and Control, 2021). The strain isolation step is necessary to demonstrate that both genes are present in the same strain. Indeed, the major challenge for EHEC identification based on *stx* and *eae* genes detection is that these genes are located on mobile genetic elements, and can be carried by non-pathogenic *E. coli* strains simultaneously present in the food matrix, as well as other *Enterobacteriaceae* (Herold et al., 2004) or even free bacteriophages (Imamovic et al., 2009). The high rates of unconfirmed presumptive positive results observed in food safety tests are a global challenge for the regulatory agencies and industry quality control laboratories performing STEC testing (Delannoy et al., 2016, 2022). It remains a desirable goal for the industry and decision makers to develop cost-effective sensitive detection tests that can guaranty the highest level of food safety. Our objective here was to refine the EHEC diagnostic systems for better identification and characterization of highly pathogenic STEC from any kind of food samples. This work was based on the hypothesis that the co-occurrence of the *stx* and *eae* genes in the same genome would imply the presence of other (variable) genes and should create complex genetic signatures. We took advantage of a Genome Wide Association Study program (GWAS) to explore a large number of *E. coli* assemblies available from public databases (Franz et al., 2014) and generated a complex matrix summarizing presence and absence for groups of orthologuous genes. Machine learning (ML) methods perform admirably in detecting predictive patterns hidden within high dimensional data (Lupolova et al., 2016; Moradigaravand et al., 2018). Supervised learning was used to create ML models that can precisely predict the co-occurrence of *stx* and *eae* genes in a genome or an assembly. After testing on simple *in silico* mixtures of strains, we successfully applied these models on long-read metagenomic sequencing data of artificially *eae*-positive STEC contaminated raw milk samples.

## 2. Materials and methods

### 2.1. Genomic data collection

Available *E. coli* genomes (*n* = 31,230) were retrieved from the GenBank database during the database construction. Based on the genome sequence completeness (full *E. coli* genomes were included in priority), the country of isolation and the *E. coli* pathotype, 1,425 genomes were selected to maximize the diversity. Sixty-eight additional genomes sequenced and assembled in a previous study by Jaudou and colleagues (Jaudou et al., 2023), were added to reach a total of 1,493 genomes. The genome accession numbers and the metadata associated with the selected genomes are reported in Supplementary Table 1. All the genomes were screened against a custom database (Available at https://github.com/fabgenomics/ML_EHEC) containing all the *stx* subtypes, *eae* and O-group genes using abricate v1.0.1. (https://github.com/tseemann/abricate). The phylogroup of each genome was determined using the EzClermont phylotyping tool available at https://github.com/nickp60/EzClermont.

### 2.2. Genome annotation and classification

The selected genomes were annotated using the rapid prokaryotic genome annotation software prokka v1.13.3 (Seemann, 2014) using the proteins option with the reference genome of *E. coli* O157:H7 str. Sakai (NC 002695.2) (Hayashi et al., 2001). Resulting General Feature Format (.gff) files were processed through a Pangenome analysis pipeline using panaroo v1.2.7 with –clean-mode strict, –remove invalid-genes and –merge paralogs options (Tonkin-Hill et al., 2020). Panaroo collapses genes into putative families with a family sequence identity level of 70% by default and creates groups. The gene_presence_absence.Rtab table provided by the panaroo output contains all the groups and genes and the presence/absence information from each genome. These groups and genes were renamed using a custom script (Available at https://github.com/fabgenomics/ML_EHEC) in which we used the information in the panaroo output file matrix (gene_presence_absence.csv available at https://doi.org/10.5281/zenodo.7129021) to retrieve the corresponding locus tag number from the *E. coli* O157:H7 Sakai strain annotation (ECs number) relative to the group or the gene (CDS). Only groups renamed with the locus tag number by our custom script were retained for further analysis. We created a CDS presence/absence matrix (ECs_presence_absence.csv available at https://doi.org/10.5281/zenodo.7129021), by adding a new column based on the pathotype of each genome. Genomes that were found to be *stx*+/*eae*+ were assigned to the EHEC pathotype and noted 1 in the table and all the other genomes (STEC for *stx*+/*eae*-, EPEC for *stx*-/*eae*+ and the abbreviation COM was used for *stx*-/*eae*-) were considered non-EHEC pathotype and noted 0. A phylogenetic tree was reconstructed using IQtree v2.0.3 (Minh et al., 2020) with the Generalised Time Reversible (GTR) model on the core genome alignment produced by panaroo. The tree was annotated with the molecular serogroups using the CLC genomic workbench v21 (QIAGEN, Aarhus, Denmark).

### 2.3. Machine learning model training and evaluation

**Figure 1:**
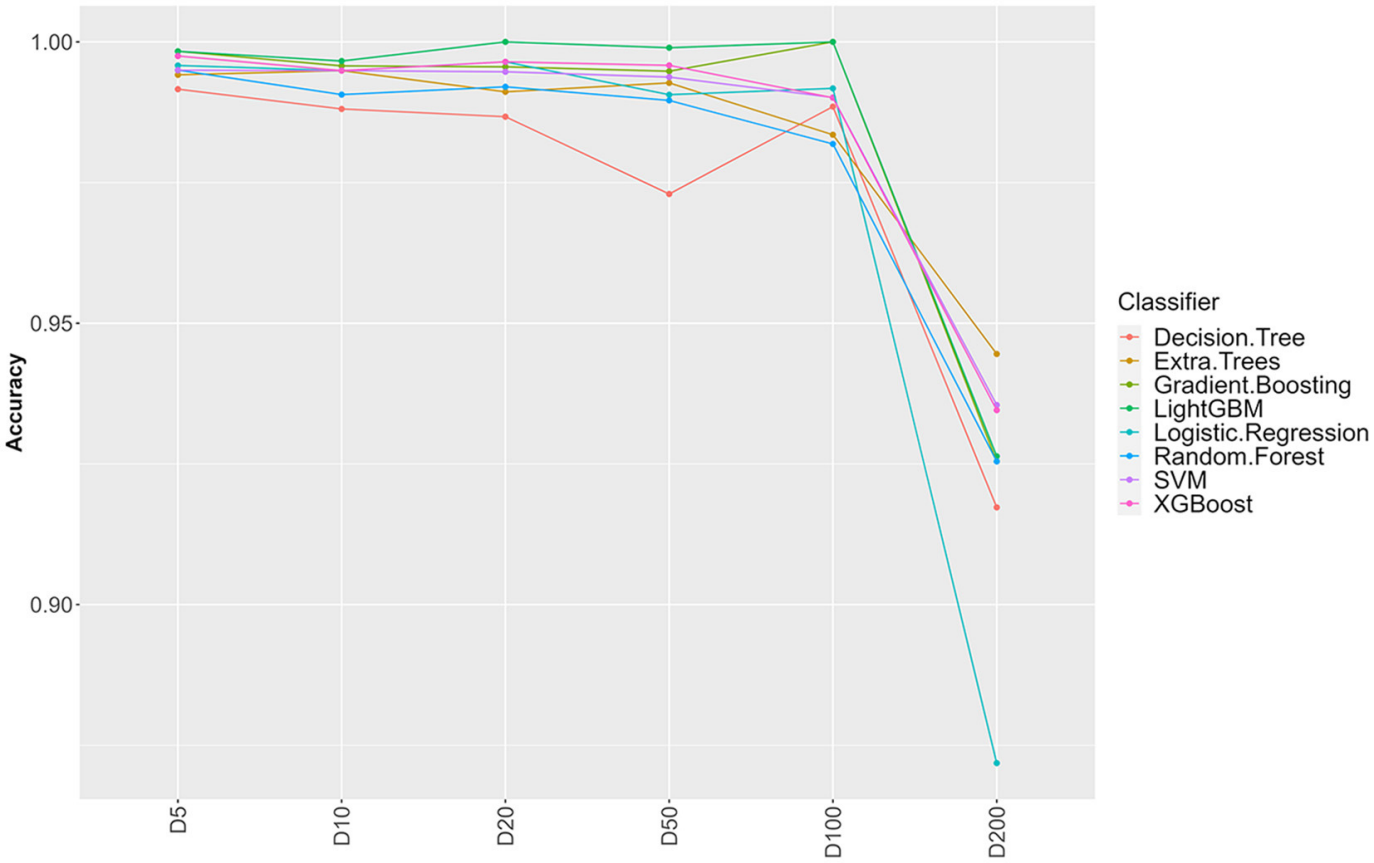
Influence of sample clustering on classification performance. For the different classification algorithms, the average accuracy (over 10 fold cross validation) is reported for different values for the clustering distance *D* (cluster sizes: D5 = 1,489, D10 = 1,470. D20 = 1,410, D50 = 1,203, D100 = 756, D200 = 137).

**Figure 2:**
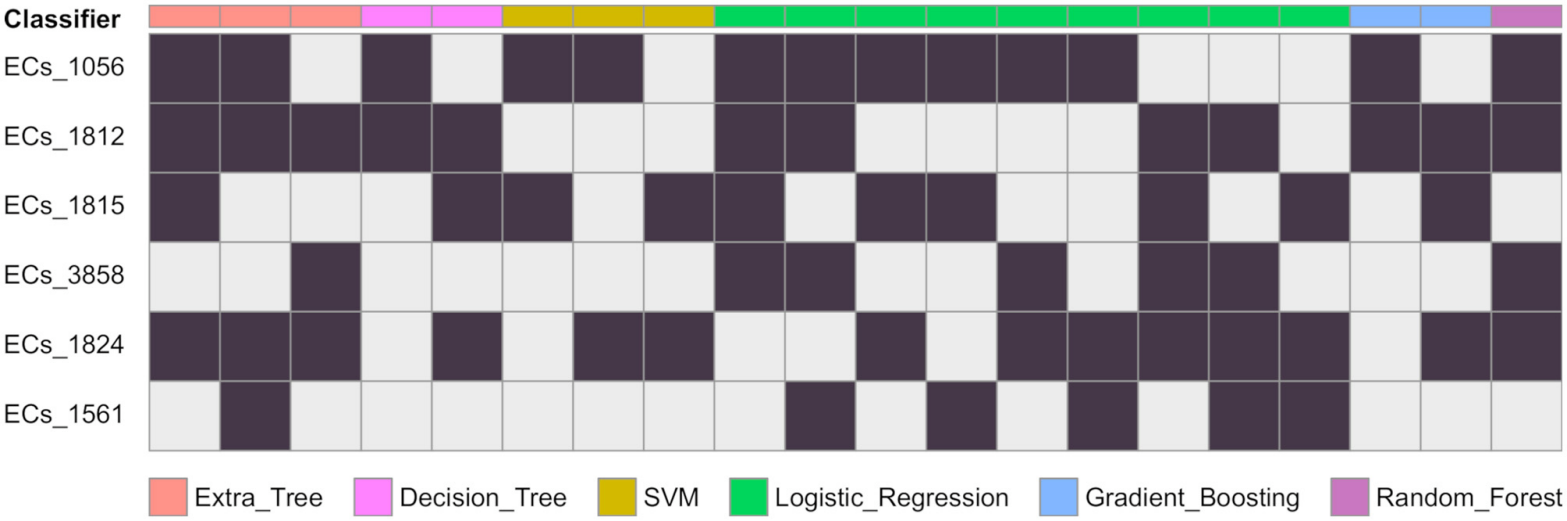
Gene combinations that are predicted as EHEC. For each classifier, the combination of genes predicting the EHEC pathotype are given in columns. A black cells means the gene is present and a grey cell that there is no constraint. For instance a decision tree predicts the EHEC pathotype if either ECs_1056 and ECs_1812 are present (column 4), or if ECs_1812, ECs_1815, and ECs_1824 are present (column 5). The number of possible combinations for each of the classifier are ET: 3, DT: 2, SVM: 3, LR: 9, GB: 2, RF: 1.

**Figure 3:**
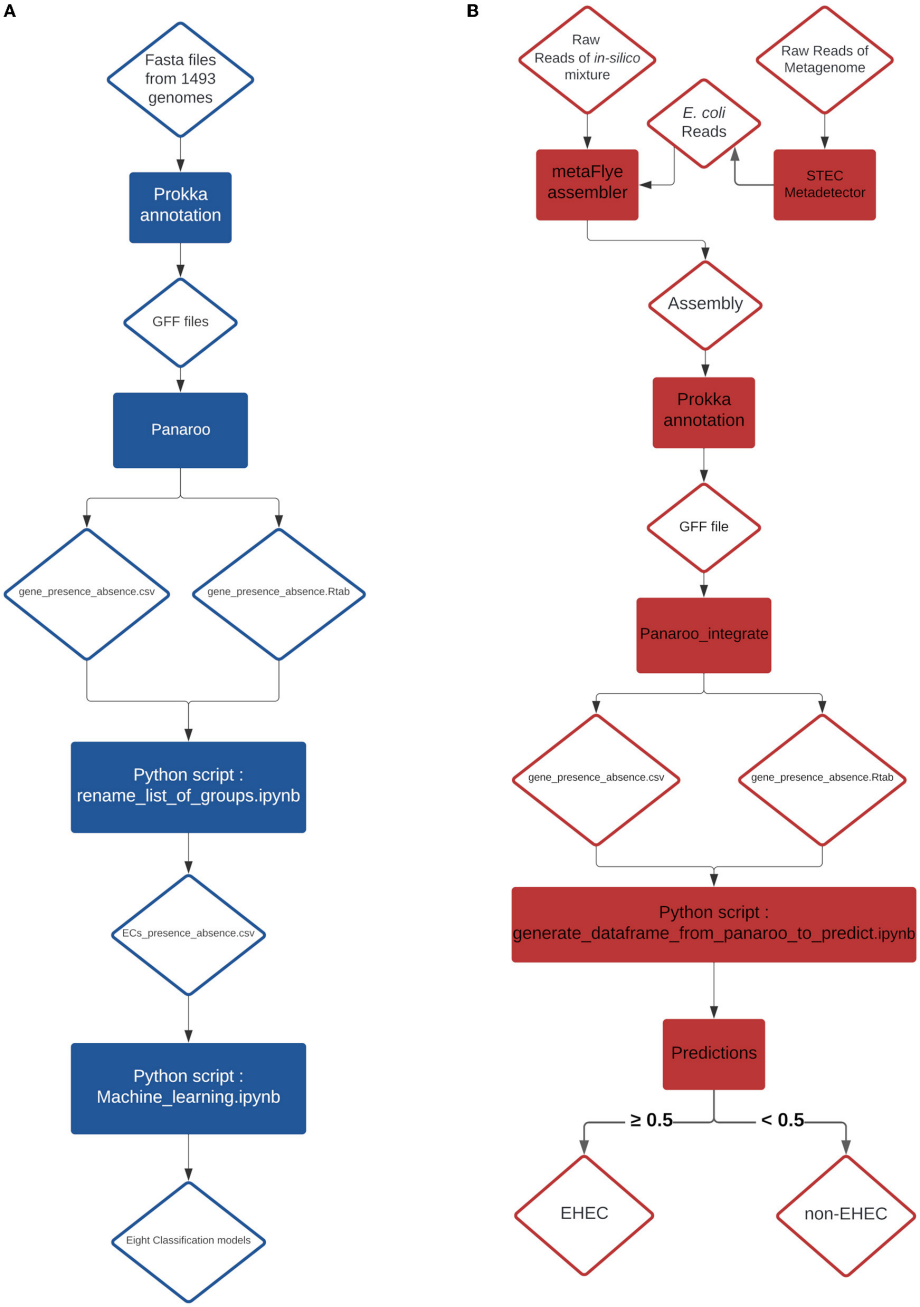
**(A)** Training pipeline for the eight models. **(B)** Pipeline of the prediction on *in-silico* mixtures and artificially contaminated raw milk.

Before evaluating the performance of the different classifiers, the CDS presence/absence matrix was filtered on non-informative features. Any CDS with less than 10% variance on its presence/absence vector was removed, as these loci do not contain useful information to test machine learning algorithms. This step removed 3,603 CDS, resulting in a dataset with 1,178 CDS. Then, to avoid possible data leakage between training and testing datasets, we grouped the samples based on their similarity. For each pair of samples, their CDS presence/absence vector was used to compute a hamming distance (*i.e*. the number of differences). Any two samples with a hamming distance lower than or equal to *D* were allocated to the same cluster. We considered possible values for *D* of 5, 10, 50, 100 and 200. Note that the resulting clusters have extremely homogeneous pathotypes (at *D* = 100, only one cluster consists of a mixture of EHEC and non-EHEC samples). The cluster table is available at https://github.com/fabgenomics/ML_EHEC. The main dataset (*n* = 1,493) was then subsampled to keep only one genome from each cluster. Then, each subsampled dataset was randomly split using 80% of the samples for training/validation and the remaining 20% for testing. The train_test_split module from Sklearn was used, with stratify option to control the proportion of EHEC in both datasets. Eight classification algorithms were trained on each dataset using 10-fold Cross-Validation: Decision Tree, Extra Tree, Gradient Boosting, LGBMClassifier, Logistic Regression, Random Forest, XGBClassifier and Support Vector Machine. The evaluation metric used was the function cross_val_score from the sklearn library. For all the cross-validation scores (10 folds *i.e*. 10 scores), the mean accuracy was calculated (Supplementary Table 2 and Figure 1). Further analysis were performed using a distance *D* = 100 for clustering (dataset Cluster-D100). This implies that two samples in this dataset differ by at least 8.4% (100/1178 CDS) of their gene content. A module from Sklearn library v0.23.1 (RandomUnderSampler) was used to select randomly the same amount of non-EHEC genomes to be equal to the number of remaining EHEC genomes from the cluster analysis. The Cluster-D100 dataset was randomly split with a ratio of 80/20% for training and testing datasets respectively and the stratify option. Eight classification algorithms were used to select the most important features with the SelectFromModel library from Sklearn. The most important CDS are listed in Table 1. We arbitrarily chose to select the six most important features to create a new reduced dataset. With this resulting matrix, hyper-parameter tuning was done on each of the eight models using RandomizedSearchCV and GridSearchCV (scoring on roc_auc metric) and cross-validation steps (*n* = 5) when the option was available. Finally, each classifier was retrained with its best hyper-parameter and evaluated on the testing dataset previously set aside (accuracy, precision, recall and F1-score are obtained using the classification_report from Sklearn, see Supplementary Table 3). To understand which gene combination led to the prediction of the EHEC pathotype, we generated all 2^6^ = 64 combinations of the six genes presence/absence and computed, for each ML model, the probability of the EHEC pathotype. We then kept the cases where the probability was ≥0.7 and transformed the set of gene combinations into simplified boolean expressions (using a boolean Algebra Solver). The results are reported on Figure 2 Charts of the training pipeline and the prediction pipeline are presented in Figures 3A, B, respectively.

**Table 1:** Top six most important features extracted from the training of the eight classification models.

**Rank**	**Locus_tag**	**Gene ID**	**Gene name**	**Encoded protein**	**Number of models using the gene for EHEC prediction**
1	ECs_1056	62675958	-	Phage excisionase	8
2	ECs_1812	912909	*nleA/espI*	T3SS secreted effector NleA/EspI	7
3	ECs_1824	912888	*nleG*	T3SS secreted effector NleG	5
4	ECs_3858	916318	*nleE*	T3SS secreted effector NleE	4
5	ECs_1815	912903	*nleF*	T3SS secreted effector NleF	4
6	ECs_1561	913337	*espN*	T3SS secreted effector EspN	4

The Gene ID of each locus is provided, and when known, the gene name as well.

### 2.4. Evaluation of the eight models on *in silico* mixtures of *E. coli*

From a previous study conducted by Jaudou et al. (2023), raw ONT MinION reads from two STEC (ECA279 (O174:H2) = SRR18191627 and 97HMPL652 (O110:H9) = SRR18191587), one *stx*-negative *eae*-positive *E. coli* (2142-O103 (O103:H25) = SRR18191529), one *eae*-positive STEC (*E. coli* 12-1 (O157:H7) = SRR18191640) and one commensal *E. coli* [i.e., negative for both *stx* and *eae* (NC809 (O41:H7) = SRR18191621)] were collected. One *stx*-negative *eae*-positive *E. coli* strain (KK072/05 - O156:H8) was newly sequenced during this study following the same protocol described by Jaudou *et al*. (Jaudou et al., 2023) and the raw ONT MinION reads were deposited to the NCBI database under the number SRX18376762. Raw reads were subsampled using Rasusa v0.6.0 (Hall, 2022) with 5.5Mb for the target number of bases and 10, 20, 30, 40 and 50x for the coverage. From these subsampled reads, twenty-five *in silico* mixtures were generated by concatenating into a 1:1 ratio the same coverage of subsampled reads. Details of the different mixtures are presented in the Table 2. From these mixtures, an assembly was generated using metaFlye v2.9-b1768 (Kolmogorov et al., 2020) with nano-raw and meta parameters. The resulting assemblies were annotated with the same parameters as described in the Genome annotation and classification paragraph (Section 2.2). The produced GFF file of each annotation was integrated individually in the pangenome graph generated with the 1,493 genomes using the panaroo-integrate command from the panaroo program. From the new gene_presence_absence.csv and the gene presence absence.Rtab generated by the panaroo-integrate command, the row corresponding to the added mixture was extracted using a newly developed python script (Available at https://github.com/fabgenomics/ML_EHEC) to reconstruct the CDS presence/absence table. We extracted only the features required for the tested model and when a feature was absent, we created it and introduced a 0 value. The data extracted were used to perform the predictions (Figure 3B). The predict_proba method from all the algorithms was used to estimate the probability that the sample is an EHEC.

**Table 2:** Prediction of the class probabilities on the 25 generated mixtures of pure *E. coli* cultures.

**Strains and genome coverage used for *in-silico* mixture**	*E. coli* Pathotype mixture^*^	Class^**^	**LGBM**	**LR**	**DT**	**XGB**	**RF**	**SVM**	**GB**	**ET**
ECA279 + NC809 10x	STEC-COM	0	0.00	0.02	0.00	0.00	0.04	0.01	0.02	0.03
ECA279 + NC809 20x	STEC-COM	0	0.00	0.02	0.00	0.00	0.04	0.01	0.02	0.03
ECA279 + NC809 30x	STEC-COM	0	0.00	0.02	0.00	0.00	0.04	0.01	0.02	0.03
ECA279 + NC809 40x	STEC-COM	0	0.00	0.02	0.00	0.00	0.04	0.01	0.02	0.03
ECA279 + NC809 50x	STEC-COM	0	0.00	0.02	0.00	0.00	0.04	0.01	0.02	0.03
97HMPL652 + 2142-O103 10x	STEC-EPEC	0	0.22	0.27	0.00	0.27	0.46	0.21	0.02	0.47
97HMPL652 + 2142-O103 20x	STEC-EPEC	0	0.22	0.27	0.00	0.27	0.46	0.21	0.02	0.47
97HMPL652 + 2142-O103 30x	STEC-EPEC	0	0.98	0.96	1.00	1.00	0.87	1.00	0.98	0.88
97HMPL652 + 2142-O103 40x	STEC-EPEC	0	0.73	0.72	0.75	0.75	0.73	0.88	0.98	0.77
97HMPL652 + 2142-O103 50x	STEC-EPEC	0	0.73	0.72	0.75	0.75	0.73	0.88	0.98	0.77
97HMPL652 + KK072/05 10x	STEC-EPEC	0	0.05	0.20	0.00	0.01	0.26	0.01	0.02	0.25
97HMPL652 + KK072/05 20x	STEC-EPEC	0	0.05	0.20	0.00	0.01	0.26	0.01	0.02	0.25
97HMPL652 + KK072/05 30x	STEC-EPEC	0	0.05	0.20	0.00	0.01	0.26	0.01	0.02	0.25
97HMPL652 + KK072/05 40x	STEC-EPEC	0	0.05	0.20	0.00	0.01	0.26	0.01	0.02	0.25
97HMPL652 + KK072/05 50x	STEC-EPEC	0	0.05	0.20	0.00	0.01	0.26	0.01	0.02	0.25
Ecoli12-1 + NC809 10x	EHEC-COM	1	1.00	0.99	1.00	1.00	0.95	1.00	0.98	0.98
Ecoli12-1 + NC809 20x	EHEC-COM	1	1.00	0.99	1.00	1.00	0.95	1.00	0.98	0.98
Ecoli12-1 + NC809 30x	EHEC-COM	1	1.00	0.99	1.00	1.00	0.95	1.00	0.98	0.98
Ecoli12-1 + NC809 40x	EHEC-COM	1	0.97	0.92	0.75	0.75	0.82	0.88	0.98	0.86
Ecoli12-1 + NC809 50x	EHEC-COM	1	1.00	0.99	1.00	1.00	0.95	1.00	0.98	0.98
ECA279 + Ecoli12-1 10x	STEC-EHEC	1	0.97	0.92	0.75	0.75	0.82	0.88	0.98	0.86
ECA279 + Ecoli12-1 20x	STEC-EHEC	1	0.97	0.92	0.75	0.75	0.82	0.88	0.98	0.86
ECA279 + Ecoli12-1 30x	STEC-EHEC	1	0.97	0.92	0.75	0.75	0.82	0.88	0.98	0.86
ECA279 + Ecoli12-1 40x	STEC-EHEC	1	0.97	0.92	0.75	0.75	0.82	0.88	0.98	0.86
ECA279 + Ecoli12-1 50x	STEC-EHEC	1	0.97	0.92	0.75	0.75	0.82	0.88	0.98	0.86

^*^EHEC, enterohemorrhagic *E. coli*; STEC, Shiga toxin-producing *Escherichia coli*; EPEC, enteropathogenic *E. coli*; COM, commensal *Escherichia coli*.

^**^Non-EHEC = 0; EHEC = 1.

LGBM, LGBMClassifier; LR, Logistic Regression; DT, Decision Tree; XGB, XGBClassifier; RF, Random Forest; SVM, Support Vector Machine; GB, Gradient Boosting; ET, Extra Tree.

### 2.5. Evaluation of the eight models on experimentally-contaminated raw milk

Eight metagenomes from artificially contaminated raw milks described in a previous study (Jaudou et al., 2022) were downloaded from the Genbank public database (Table 3). The estimated level of contamination was 0.5x10^3^, 0.5x10^2^ and 0.5x10^1^ CFU.mL^−1^ of EHEC O26 plus one EHEC-free milk. Raw reads were processed using the STECmetadetector pipeline developed by Jaudou et al. (2022) available at https://gitlab.com/Bfr_bioinformatics/STECmetadetector and the extracted *E. coli* reads were assembled using metaFlye v2.9-b1768 with the same parameters as described in Section 2.4. The resulting assemblies were annotated with the same parameters as described in the Genome annotation and classification paragraph (Section 2.2). The resulting GFF file was treated with the same process than the *in-silico* mixture GFF file and the EHEC predictions were performed, as described in Section 2.3 (Figure 3B).

**Table 3:** Prediction of the class probabilities on the milk metagenomes.

**Strain**	Accession number^*^	EHEC spiking level^**^	Class^***^	**LGBM**	**LR**	**DT**	**XGB**	**RF**	**SVM**	**GB**	**ET**
4712-O26	SRR19090780	0.5x10^3^	1	1.00	0.99	1.00	1.00	0.95	1.00	0.98	0.98
6423-O26	SRR19090775	0.5x10^3^	1	1.00	0.99	1.00	1.00	0.95	1.00	0.98	0.98
4712-O26	SRR19090792	0.5x10^2^	1	1.00	0.99	1.00	1.00	0.95	1.00	0.98	0.98
6423-O26	SRR19090774	0.5x10^2^	1	1.00	0.99	1.00	1.00	0.95	1.00	0.98	0.98
4712-O26	SRR19090778	0.5x10^1^	1	1.00	0.99	1.00	1.00	0.95	1.00	0.98	0.98
6423-O26	SRR19090772	0.5x10^1^	1	1.00	0.99	1.00	1.00	0.95	1.00	0.98	0.98
6423-O26	SRR19090769	0.5x10^1^	1	1.00	0.99	1.00	1.00	0.95	1.00	0.98	0.98
EHEC-neg	SRR19090777	0	0	0.02	0.15	0.00	0.03	0.18	0.21	0.02	0.13

^*^From Jaudou et al. (2022).

^**^CFU.mL^−1^.

^***^Non-EHEC = 0; EHEC = 1.

LGBM, LGBMClassifier; LR, Logistic Regression; DT, Decision Tree; XGB, XGBClassifier; RF, Random Forest; SVM, Support Vector Machine; GB, Gradient Boosting; ET, Extra Tree.

## 3. Results

### 3.1. *Escherichia coli* pathotype assignation based on genomic information

**Figure 4:**
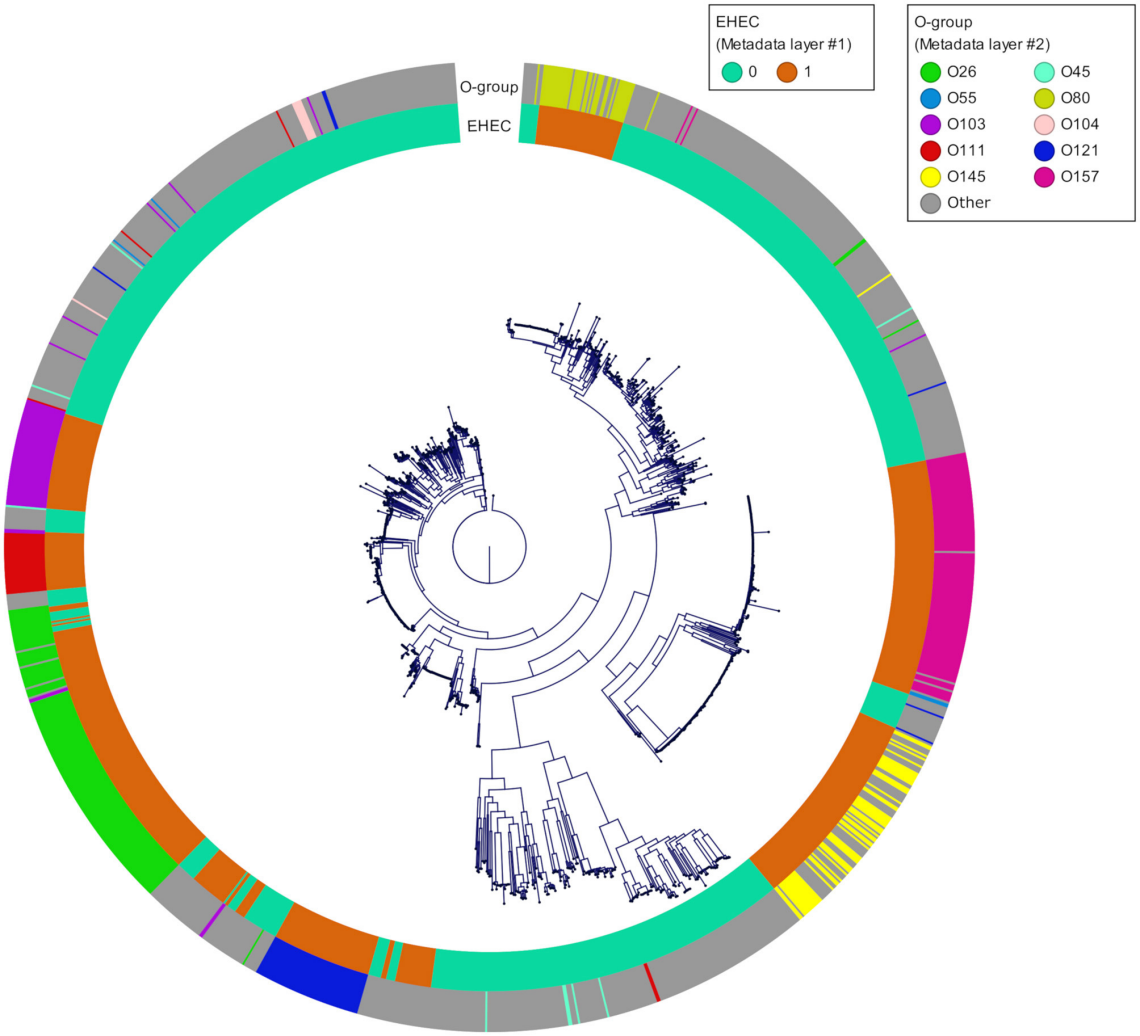
Phylogenetic tree of the 1,493 genomes reconstructed from the core genome alignment. The inner ring corresponds to the pathotype of each strain determined based on *stx* and *eae* presence/absence (0 for all non-EHEC and 1 for the EHEC). The outer ring corresponds to the top 10 O-groups.

To take advantages of ML to find patterns and preserve its generalization potential, we constituted an *E. coli* database for which we selected in priority complete *E. coli* genomes with a minimum of required metadata (origin, isolation date and location) and verified their pathotypes (*stx* and *eae* genes presence). A total of 1,493 genomes were downloaded from the GenBank database. The geographic origin of the strains was 33, 26, 23, 7, and 3% from Europe, Asia, America, Africa and Oceania, respectively and the 8% remaining were missing the country of origin. During the genome selection, we tried to respect an equal proportions of *stx*/*eae*-positive strains (i.e., EHEC) and non-EHEC strains based on the metadata provided. Genomes simultaneously positive for at least one *stx* gene and the *eae* gene (*n* = 632) were assigned to the EHEC group. The other genomes (*n* = 861) were assigned to the non-EHEC group (Available at https://doi.org/10.5281/zenodo.7129021). In addition, the custom database was reporting all the O-group sequences so that the serogroup information, in particular the most frequent EHEC serogroup, was available (Supplementary Table 1). The top seven most represented serogroups were O26 (*n* = 160), O157 (*n* = 126), O103 (*n* = 66), O121 (*n* = 61), O145 (*n* = 60), O111 (*n* = 36), and O45 (*n* = 9). These serogroups comprised mostly EHEC strains with 126 EHEC O26 strains, 124 EHEC O157 strains, 57 EHEC O103 strains, 55 EHEC O121 strains, 59 EHEC O145 strains, and 31 EHEC O111 strains. The phylogroup analysis showed that the diversity of the species is well represented. The phylogeny of the final dataset is illustrated in Figure 4.

### 3.2. Generation of the input dataset

From 37,380 groups generated by panaroo, 13,952 remained with an ECs annotation. Some ECs were duplicated in the table due to the genetic diversity of some genes and the panaroo identity level threshold. We aggregated the results for these genes, which resulted in 4,780 unique CDS and kept the presence/absence information. Before splitting the ECs presence/absence table (Available at https://doi.org/10.5281/zenodo.7129021), we first selected CDS with enough variation between the samples (see Section 2). This filtering step removed 3,602 CDS resulting in a dataset containing 1,178 CDS. To avoid optimistic performance estimates that would result from near duplicate samples both in training and testing sets, we performed a clustering based on gene content similarity (see Section 2). Clustering at distance *D* ensures that two clusters in the dataset have at least *D* genes that are different. We evaluated the change in accuracy for increasing values of *D*, starting from 5 and up to 200 (Figure 1). Up to *D* = 100 (8.4% of the CDS), the accuracy of all classifiers remains very high (above 97%). This indicates that robust information can be extracted from gene profiles to predict EHEC pathotype with high accuracy. The performances drop by around 5–10% for *D* = 200 for an accuracy of around 93%. While still high, this decrease could be attributed to the low number of clusters that remain for prediction (*n* = 137, 15 EHEC and 122 non-EHEC). From these results, a value of *D* = 100 was chosen to build the dataset for further analysis, as it combines both good performance with a sufficient sample size (*n* = 756). However, this dataset was imbalanced. To avoid overfitting problems, we subsampled the non-EHEC class to be equal to the EHEC class and ended with a balanced dataset (see Section 2). The matrix was then randomly split into a training dataset containing 80% of the genomes used in the study (*n* = 139) with 69 non-EHEC and 70 EHEC. The testing dataset contained the remaining 20% (*n* = 35) with 18 non-EHEC and 17 EHEC.

### 3.3. CDS selection using eight ML classifiers

We conducted a comprehensive analysis of the top features extracted from the training of eight classification models, and the results are summarized in Table 1. The table shows the top six most important features, ranked based on the number of times they were used by the models. The most important feature was found to be ECs_1056, which corresponds to a phage excisionase gene. This feature was used by eight of the models. The second most important feature was ECs_1812, which corresponds to the *nleA/espI* gene, coding for a type III secretion system (T3SS) secreted effector protein, and was used by seven of the models. The third most important feature was ECs_1824, which corresponds to the *nleG* gene, which encodes another T3SS secreted effector protein, and was used by five of the models. The remaining features, namely ECs_3858, ECs_1815, and ECs_1561, were used by four models each, and correspond to the *nleE, nleF*, and *espN* genes, respectively, all of which encodes T3SS secreted effector proteins. Multiple classifiers achieved near perfect performance when evaluated on the *D*100 dataset. To better understand which combinations of genes contribute to the prediction of the EHEC pathotype, we generated all 64 (2^6^) genes presence/absence profiles and recorded in which case one of the models predicted an EHEC pathotype with confidence (Figure 2 and Section 2). This confirms that some genes, such as ECs_1056 (phage excisionase) and ECs_1824 (*nleG*) have an importance (they are needed in the majority of the predictions). Decision Tree and Gradient boosting learned the same decision rule: “ECs_1812 (*nleA*) and (ECs_1056 or (ECs_1815 (*nleF*) and ECs_1824))”. The SVM classifier predicts EHEC for any two combination of ECs_1056, ECs_1815, and ECs_1824. All those classifiers can predict an EHEC pathotype with as little as two genes. On the other hand, Extra tree and logistic regression make predictions involving three to four genes, showing that they can have different sensitivity.

### 3.4. Performance of the eight models on the selected features

Supplementary Table 3 shows the evaluation metrics obtained by training the eight classifiers using the selected six features: ECs_1056, ECs_1812, ECs_1824, ECs_3858, ECs_1815, ECs_1561. All classifiers achieved high accuracy scores, ranging from 0.97 to 1.00, indicating a good performance in predicting the target variable. Logistic Regression, Extra Trees XGBCLassifier, LGBMClassifier, Decision Tree, and SVM achieved perfect accuracy scores of 1.00, while Random Forest and Gradient Boosting achieved a slightly lower accuracy score of 0.97. Extra Tree and Random Forest achieved a precision of 0.94 on the EHEC class and a recall of 0.94 on the non-EHEC class. All the other classifiers achieved perfect precision, recall, and F1-scores, indicating that the selected features were informative and sufficient to discriminate perfectly between the two classes.

### 3.5. EHEC prediction on *in silico E. coli* mixtures

We first tested the ability of the different models to predict the presence of an EHEC strain in a mixture of *E. coli* strains. For this purpose, *in silico* mixtures of raw MinION reads were assembled. Assemblies produced using meta-Flye ranged from 5.63 to7.94 Mb (mean = 6.57 Mb) and the number of contigs from 80 to 155 (mean = 106) (Supplementary Table 4). In all mixtures of *E. coli* strains, the assembly size was longer than a normal *E. coli* assembly (4.8–5.5 Mb). Shorter assemblies were produced by meta-Flye with the STEC-COM mixture (5.63–6.16 Mb). On the contrary, the EHEC-COM mixture produced longer assemblies (7.35–7.94 Mb). The eight models were then used to perform predictions on the *in silico* mixtures (Table 2). Predictions of the pathotype ranged from 0 to 1 and the average prediction from 0.01 to 0.99. The cut-off for binary classification is usually set to 0.5. Above or equal to this cut-off value the presence of an EHEC is predicted, and below a non-EHEC is predicted. This cut-off of 0.5 was used to report the results presented in this study. The eight classifiers were able to predict the correct class 22 times over 25 predictions (88%). However, all models incorrectly predicted the presence of an EHEC three times for the STEC-EPEC mixture with the strain 97HMPL652 and 2142-O103 for a coverage of 30x, 40x and 50x, respectively. For all non-EHEC containing mixtures, the higher value was 0.47 for the same STEC-EPEC mixture with the Extra Tree classifier (Table 2). For the EHEC class the lower value was 0.75 for the EHEC-COM mixture and the STEC-EHEC mixture with the Decision Tree classifier and the XGBClassifier. Taken together, these data indicate that all the classifiers were able to predict with high confidence the presence of an EHEC in *E. coli* mixtures that combine different *E. coli* pathotypes. Only three false positives were predicted for the most difficult mixture combining a STEC and an EPEC.

### 3.6. EHEC prediction on artificially-contaminated raw milk

We then tested the performance of the eight models on complex mixtures using artificially contaminated raw milk. A bioinformatic pipeline called STECmetadetector developed by Jaudou et al. (2022), was used to specifically extract *E. coli* reads from raw milk samples artificially contaminated with an O26 EHEC strain (from 0 to 500 CFU.mL^−1^). The *E. coli* reads were assembled using the same method as described in Section 2 (paragraph 2.5). Assemblies ranged from 5.2 Mb to 6.83 Mb (mean = 5.95 Mb) and the numbers of contigs from 5 to 62 (mean = 15). The eight classifiers were used to predict the pathotype of artificially contaminated raw milks (Table 3). All models were able to accurately predict the EHEC pathotype in the sample with high confidence, at the three contamination levels tested, regardless of the strain used for the artificial contamination. Notably, the raw milk used for spiking with the 6423-O26 strain was naturally containing commensal *E. coli* of serotype O185:H2 and O8:H19 (Jaudou et al., 2022). Predictions of the pathotype ranged from 0.95 to 1 for the class EHEC for all contamination levels. The negative control (a non-contaminated raw milk), was classified accurately as non-EHEC by all eight classifiers with the higher value of 0.21 for the SVM (Table 3).

## 4. Discussion

The correct detection and identification of highly pathogenic STEC from food remains challenging. Conventional detection methods based on the detection of the *stx* and *eae* genes (as well as genes from the most frequent serogroups) require an isolation step to ensure the correct characterization of the strain. Detection of EHEC in food samples based on the presence of a small number of additional genes that are more specifically associated with strains possessing simultaneously the *stx* and *eae* genes would represent a significant improvement for screening food samples (Delannoy et al., 2016, 2022). With such an approach, the number of presumptive positive samples that require further investigation by isolation and genotypic characterization can be reduced by around 50% (Delannoy et al., 2016, 2022), allowing to save money and time. Still, the amount of unconfirmed presumptive positive samples may be a problem for both the food industry and the decision maker. In a previous study, we showed that long-read metagenomics was efficient in identifying *eae*-positive STEC strains from complex matrices such as raw milk in an isolation-independent way (Jaudou et al., 2022). However, we have highlighted that the presence of multiple *E. coli* strains may hinder the identification of the *eae*-positive STEC due to the assembly-based approach used. We wanted to continue exploring the potential of long-read metagenomics and take full advantage of ML algorithms by applying them to predict the presence of an EHEC strain directly from *E. coli* reads assembly, even in the presence of multiple *E. coli* strains.

As of February 2021, 31,230 *E. coli* genomes were available in the NCBI Genbank database, around 10% of which are genomes of O157:H7 strains. To build our database, we downloaded complete *E. coli* genomes as well as some scaffolded genomes that contained accompanying metadata, while taking care of having the top 10 EHEC serotypes represented, as well as less frequent ones (Figure 4). Because the geographical distribution of certain clones may be skewed, we also included strains originating from all continents. During the genome selection, our objective was to obtain a database constituted at 50% of EHEC genomes (targets) and 50% of non-EHEC genomes (non-targets). When selecting the non-EHEC genomes we were careful to include various pathotypes such as EPEC (*eae*+ only), STEC (*stx*+ only), commensals (*stx* and *eae* negative) and some Extra-intestinal pathogenic *E. coli* (ExPEC) strains. Despite the size of our database and the precautions we took to build it, our final dataset after dereplication, was composed of 87 EHEC and 87 non-EHEC. We originally included large numbers of genomes for each of the top 10 serotypes observed in clinical cases worldwide in order to be representative of the frequency of isolation of the various serotypes. However, the diversity within each serotype appears limited. Indeed, several studies on various EHEC serotypes have shown that even the most diverse ones (in terms of SNPs) show a high degree of synteny and collinearity between isolates of different clades or lineages (Dallman et al., 2015; Ogura et al., 2017; Nishida et al., 2021). Also, the pool of genes included in the dataset is the pool present in the Sakai annotation. Therefore, by reducing the available pangenome and increasing the probability for each strain to possess one version of each CDS, we increased the similarity between the genomes in the dataset. To avoid possible data leakage, we chose to group all the genomes that had less than 100 genes difference in their repertoire (8.4% of the genes considered). This is a drastic filtering step, but it is, to our knowledge, the most reliable to avoid reporting biased performance estimates.

One of the first choices when designing the pipeline is whether to use raw reads or assembled data. Initial tests showed that performing the annotation directly on long reads generated a very large amount of data that was computationally too intensive for the downstream processing (not shown). Based on these results we chose to work with assemblies and used the Flye long-read assembler with the metagenome option in order to deal with highly non-uniform coverage, in particular with low level artificially-EHEC contaminated milks. An early step of the pipeline consists in the annotation of the assembled genomes. The advantage of the annotation software used, prokka, is that a reference genome can be used to standardize the annotation. In our case, we used the O157:H7 Sakai genome as reference over the K12 *E. coli* reference genome because it is an EHEC carrying around 20% more integrated genomic elements than K12 *E. coli*, like pathogenicity islands and phages (Hayashi et al., 2001). To generate the first matrix of gene presence/absence we chose panaroo among GWAS programs such as Roary (Page et al., 2015), PIRATE (Bayliss et al., 2019), or PPanGGoLiN (Gautreau et al., 2020) because it offers the possibility to add one new genome to an existing pangenome graph. This feature is the keystone of our pipeline because it is very important to add the new genome into the existing pangenome graph so as not to modify the original matrix used for training the models. Panaroo collapses genes into putative families with a family sequence identity level of 70% in the default mode. During the analysis of the generated matrix, we identified locus tags that were split in different groups and regrouped them. Indeed, the allelic variability of STEC virulence genes can be important (Michelacci et al., 2016).

Other studies have used different algorithms like Support Vector Machine, Gradient Boosting or Random Forest (Lupolova et al., 2016; Njage et al., 2019; Im et al., 2021; Shaik et al., 2022) but the nature of the data and the predictive outcome were different. In this study we used the power of ML to evaluate a high number of genes (1,178 CDS). We successfully decreased the number of genes needed for EHEC presence prediction down to six genes while keeping a high accuracy. It is remarkable that none of these six genes are related to the Shiga toxins. Surprisingly, neither *stx*1 subunit A and B nor *stx*2 subunit A and B are needed to predict an EHEC. Because it is present in all EPEC strains (*eae*-positive *E. coli*, non-target) the absence of *eae* in the six genes scheme is expected. In the reduced set of selected genes, we found five Type 3 Secretion System (T3SS) effectors and a phage excisionase. The T3SS represents an important component of the *E. coli* mobile gene pool. Although the LEE carries constitutive elements of the T3SS, additional effectors are encoded by prophages inserted into the genome (Tobe et al., 2006). A large number of studies have described T3SS effectors as associated virulence markers (Coombes et al., 2008; Konczy et al., 2008; Bugarel et al., 2010a,b, 2011; Imamovic et al., 2010; Creuzburg et al., 2011). Here, the most important features identified for EHEC prediction are located on four genomic islands that harbors putative virulence factors already demonstrated to be present in EHEC strains: Sp4 (ECs 1056 / phage excisionase), Sp6 (ECs 1561 / *espN*), Sp9 (ECs 1812 / *nleA*, ECs 1815 / *nleF*, ECs 1824 / *nleG*) and SpLE3 (ECs 3858 / *nleE*) (Tobe et al., 2006; Rasko et al., 2008; Bugarel et al., 2010a,b, 2011; Delannoy et al., 2013). The *nleA* gene (ECs 1812 - Sp9), which was found to be the second most important feature in our study, has been shown to play a key role in the virulence of various pathogenic bacteria, including *E. coli* (Rasko et al., 2008). Similarly, the *nleG* gene (ECs 1824 - Sp9), which was the third most important feature, has been shown to be important for the virulence of enterohemorrhagic *E. coli* (Tobe et al., 2006). Other T3SS effectors located in these four genomic islands have previously been shown to be associated with EHEC. For example, the Sp4 genomic island also harbors *espV* (ECs 1127), which, in combination with *espK* (ECs 1568 - Sp6) have been demonstrated to be present in EHEC strains and proposed as genetic markers to reduce false-positive results in food testing (Delannoy et al., 2013, 2016). Similarly, combinations of genes from Sp9 and SpLE3 were demonstrated to be strong signatures of typical EHECs (Bugarel et al., 2010a,b, 2011). These four genomic islands are recurrently found as harboring important features with all models and were previously experimentally found associated with EHEC. This strongly suggests that these genomic islands are stably associated with both the LEE and the presence of an *stx*-phage and may have co-evolved (Guo et al., 2012). Although, the precise order of acquisition of these mobile genetic elements remains to be determined. The only incorrect EHEC predictions of the models using *in-silico* mixtures were obtained with the STEC/EPEC mixtures containing the aEPEC strain 2142-O103. Although negative for the *stx* gene this strain harbors the different genomic islands: Sp4, Sp6, Sp9, and SpLE3. It also belongs to a known EHEC serotype (O103:H25) that has been associated with an HUS outbreak (Schimmer et al., 2008). It is thus likely that this strain represents what had previously been named an EHEC-like or EHEC-LST (Bielaszewska et al., 2007; Mellmann et al., 2008; Bugarel et al., 2011), meaning that it could constitute an EHEC progeny that has lost the *stx* phage at one point. The existence of such EHEC-like strains constitute a caveat of our approach, as it is impossible for our model to distinguish a STEC/EHEC-like mixture accurately. However, from a risk management perspective, it could be beneficial to detect this kind of strains when in the presence of other STEC strains due to the potential of these EHEC-like to acquire the *stx* phage and become typical EHEC (Bielaszewska et al., 2007; Mellmann et al., 2008). Correct and timely identification of EHEC is crucial in food microbiology as well as for surveillance of STEC-mediated disease. The growing genomic sequence data offers additive information that may support the identification of discriminative EHEC markers (Kiel et al., 2018). To extend EHEC diagnostics in the post-genomic era beyond the detection of the O157:H7 and the non-O157 serogroups from the Top 7, we developed suitable pipelines that integrate high throughput sequence data, to predict with high specificity and sensitivity EHEC strains. Different combinations of discriminative genetic markers were identified and validated to target the main STEC subgroup (*eae*-positive STEC) associated with severe human infections and outbreaks worldwide. Our study is in line with recent papers showing the potential and power of GWAS and Machine Learning approaches for designing biomarkers that target foodborne pathogens (Feucherolles et al., 2021; Sévellec et al., 2022). Here, the description of these new EHEC biomarkers is the confirmation that *stx* and *eae* are not the only genetic markers that are the hallmark of EHEC, but that EHEC characterization is much more complex than the simultaneous identification of *stx* and *eae* genes. There are in fact associated factors (type III effectors are some of them as shown in this study) which, by their presence or absence, provide a fairly precise predictive model on the co-localization of *stx* and *eae* in a single strain. The new EHEC markers found using ML in our study could predict EHEC with very high accuracy in a large genome dataset and artificially contaminated raw milk metagenomes. The correct prediction of the EHEC strain while co-occurring with another *E. coli* strain at a ratio of 1:1 is remarkable. Most programs that aim at distinguishing strains from the same species relies on coverage differences (*i.e*. for assemblers and binning tools). These findings open the door for the development of new diagnostics tests for a better screening of EHEC in foods products. As long as DNA sequence-based diagnostics of mixed populations cannot resolve whether relevant markers like *stx* and *eae* genes are present in the same genome, some risk of generating false-positive results exist. Including the combination of additional EHEC-related markers like those we described here, in the detection scheme, would supports a better hazard characterization of typical EHEC.

## Data availability statement

The datasets presented in this study can be found in online repositories. The names of the repository/repositories and accession number(s) can be found in the article/Supplementary material.

## Author contributions

FV, SJ, SD, and PF conceptualized the project. PF and SD were in charge of funding acquisition. SJ downloaded the fasta from public database. SJ and M-LT did the milk artificially contaminated sequencing and assembly. FV did the *in silico* mixtures analysis, machine learning model development, database creation, and wrote the original draft. Methodology and resources by FV, SJ, SD, and HR. HR did the cluster analysis. All authors contributed to manuscript revision, read, and approved the submitted version.
